# Hydrothermally Assisted Synthesis of Porous Polyaniline@Carbon Nanotubes–Manganese Dioxide Ternary Composite for Potential Application in Supercapattery

**DOI:** 10.3390/polym12122918

**Published:** 2020-12-05

**Authors:** Javed Iqbal, Mohammad Omaish Ansari, Arshid Numan, S. Wageh, Ahmed Al-Ghamdi, Mohd Gulfam Alam, Pramod Kumar, Rashida Jafer, Shahid Bashir, A. H. Rajpar

**Affiliations:** 1 Center of Nanotechnology, King Abdulaziz University, Jeddah 21589, Saudi Arabia; iqbaljavedch@gmail.com; 2 State Key Laboratory of ASIC and System, SIST, Fudan University, Shanghai 200433, China; numan.arshed@gmail.com; 3 Department of Physics, Faculty of Science, King Abdulaziz University, Jeddah 21589, Saudi Arabia; AGAMDI@kau.edu.sa (A.A.-G.); rashida.jafer@gmail.com (R.J.); 4 Physics and Engineering Mathematics Department, Faculty of Electronic Engineering, Menoufia University, Menoufia 32952, Egypt; 5 Department of Chemistry, Faculty of Science, Islamic University of Madinah, Madinah 42351, Saudi Arabia; gulfam.alam@gmail.com; 6 Department of Chemistry, Prof. Rajendra Singh (Rajju Bhaiya) Institute of Physical Sciences for Study and Research, V. B. S. Purvanchal University, Jaunpur 222003, India; pkchemistry.2009@gmail.com; 7 Center for Ionics University of Malaya, Department of Physics, Faculty of Science, University of Malaya, Kuala Lumpur 50603, Malaysia; shahidbashirbaig@gmail.com; 8 Mechanical Engineering Department, Jouf University, Sakaka 42421, Saudi Arabia; altafrajpar@yahoo.com

**Keywords:** conducting polymer, polyaniline, MnO_2_, CNT, supercapattery, energy storage device

## Abstract

In this study, ternary composites of polyaniline (PANI) with manganese dioxide (MnO_2_) nanorods and carbon nanotubes (CNTs) were prepared by employing a hydrothermal methodology and in-situ oxidative polymerization of aniline. The morphological analysis by scanning electron microscopy showed that the MnO_2_ possessed nanorod like structures in its pristine form, while in the ternary PANI@CNT/MnO_2_ composite, coating of PANI over CNT/MnO_2_, rods/tubes were evidently seen. The structural analysis by X-ray diffraction and X-ray photoelectron spectroscopy showed peaks corresponding to MnO_2_, PANI and CNT, which suggested efficacy of the synthesis methodology. The electrochemical performance in contrast to individual components revealed the enhanced performance of PANI@CNT/MnO_2_ composite due to the synergistic/additional effect of PANI, CNT and MnO_2_ compared to pure MnO_2_, PANI and PANI@CNT. The PANI@CNT/MnO_2_ ternary composite exhibited an excellent specific capacity of 143.26 C g^−1^ at a scan rate of 3 mV s^−1^. The cyclic stability of the supercapattery (PANI@CNT/MnO_2_/activated carbon)—consisting of a battery type electrode—demonstrated a gradual increase in specific capacity with continuous charge–discharge over ~1000 cycles and showed a cyclic stability of 119% compared to its initial value after 3500 cycles.

## 1. Introduction

Due to the continuous depletion of fossil fuels and the general awareness toward environmental protection and sustainable living, there is an ongoing search for an alternative renewable energy source [1]. The problem with the renewable sources are their unavailability sometimes, i.e., the solar sources will only produce significant energy when the intensity of the sunlight is high, and the wind will only produce energy when it is blowing [2]. Thus, to utilize newer energy sources and renewable sources, batteries and supercapacitors have attracted much interest for future energy storage devices [3]. Supercapacitors have gained tremendous attention due to their peculiar properties, such as higher magnitude of power density, charge–discharge with long cyclic stability, and higher power density than conventional batteries [4,5,6]. The electrode materials in supercapacitors largely determine their performance, and the ideal electrode should possess larger specific surface area (to ensure high specific capacity because charge is stored on the surface of the electrodes), good conductivity to ensure high power density, long cyclic life and environmentally sustainability [7]. Generally, the supercapacitors are of two types, i.e., electrical double layer capacitors (EDLCs) and pseudo-capacitors [8,9]. In the electrical double layer capacitor, the capacitance arises from the electrode/electrolyte interface charge separation, while in the pseudo capacitor it is because of the fast reversible faradaic reactions that occur at the surface of the electrodes [10].

Carbon materials show EDLC and have fast charge–discharge characteristics with long cyclic stability, but in contrast they possess relatively low capacitance (100~300 F g^−1^) and energy density (<10 Wh kg^−1^) [11]. In recent years, the use of carbon nanotubes (CNTs) as EDCLs has gained attention because of properties of elevated conductivity, high surface area, long stability, mechanical strength, and knittability [12]. However, due to their low specific capacity and energy density they cannot beat the pseudocapacitors due to their inherited electrostatic surface charging mechanism. Thus, the best way to utilize CNTs is to combine them with conducting polymers and metal oxides.

Out of various conducting polymers, polyaniline (PANI) stands far apart due to its low cost, simple synthesis protocol, controllable redox states, controllable morphology and tunable conductivity [13]. Deng et al. [14] interpreted that the higher capacitance of 183 F g^−1^ of PANI@CNT in contrast to capacitance of 47 F g^−1^ for CNT is due to the pseudocapacitance provided by the uniformly coated PANI over CNT. Similarly, Sharma et al. [15] in their PANI@CNT composite showed that the specific capacitance increased manifold from 49.58 F g^−1^ for CNT to 597.82 F g^−1^ in PANI@CNT at scan rate of 2 mV s^−1^. As for metal oxides, MnO_2_ is a suitable candidate owing to its pseudocapacitive properties, wide availability, low toxicity and ease of preparation [16]. MnO_2_ possess high theoretical capacity of 1370 F g^−1^ suggesting a single-electron redox reaction of all manganese atoms, broad potential window and good electrochemical characteristics in neutral electrolyte [17]. Despite these, the specific capacitance of MnO_2_ is far less than the expected value. This might be due to its poor electronic conductivity of 10^−5^–10^−6^ S cm^−1^, low ionic diffusion constant of ~10^−13^ cm^2^ V^−1^ s^−1^, low structural stability and high particle aggregation at nanoscale [18]. The incorporation of MnO_2_ into porous materials is one of the ways to enhance its performance for effective utilization as electrode materials. Li et al. [19] showed that MnO_2_@CNT nanocomposite possessed high specific capacitance of 201 F g^−1^ with cyclic stability of over 10,000 cycles. They interpreted that the bonding of MnO_2_ and CNTs reduces the contact resistance leading to simplification of Faraday reaction for the electrochemically active species. The synergized effect of conductive CNTs and pseudocapacitive MnO_2_ leads to enhanced capacitance. Similarly, Liu et al. [20] showed high pseudocapacitive nature in PANI@MnO_2_ nanocomposite electrodes. Apart from the pseudocapacitive behavior, they interpreted that the high capacitance is due to the special nanostructure and high porosity which provides effective diffusion channels for the electrolyte ions. On the basis of the above based discussion, it can be interpreted that nanostructured porous composites of PANI, CNT and MnO_2_ can be exciting materials for the fabrication of supercapattery with enhanced performance, owing to the synergistic and addition effect of pseudocapacitance and electrical double layer capacitance.

Thus, in this work ternary composite of PANI, CNT and MnO_2_ (PANI@CNT/MnO_2_) was prepared by the combination of hydrothermal methodology and in-situ oxidative polymerization. The MnO_2_ in the reaction mixture acted as oxidant and its dissolution and penetration into PANI network resulted in the generation of porosity in the PANI chains. The porosity and penetration of MnO_2_ inside leads to good contact between the constituents, i.e., PANI, CNT and MnO_2_. The high specific capacity of 131.27 C g^−1^ along with stability of over 119% cycles holds promise for its application in future enhanced supercapattery devices.

## 2. Materials and Methods

### 2.1. Materials

Aniline and activated carbon (AC) was purchased from Sigma Aldrich. Potassium per manganate (PPs), potassium per sulphate, N-methyl-2-pyrrolidone (NMP), HCl (35%) and ethanol was purchased from Otto chemicals. The CNTs with a diameter of 10–20 nm and length ~20 µm were purchased from Hanwha Nanotech, South Korea. The water used in the experiments was deionized water.

### 2.2. Synthesis of MnO_2_, PANI, PANI@CNT and PANI@CNT/MnO_2_

MnO_2_ nanorods were prepared by hydrothermal methodology. In this protocol, 0.658 g of KMnO_4_ was dissolved in 75 mL of water and to which 1.5 mL of HCl was added. The whole system was put under continuous stirring for 15 min and thereafter the solution was charged into Teflon lined hydrothermal reactor of volume 100 mL and kept at 140 °C for 24 h [21]. The final brown color indicated successful formation of MnO_2_. Afterwards centrifugation was used to separate prepared MnO_2_, which was then washed with excess of water and ethanol and subsequently dried at 100 °C for 6 h. PANI@CNT was prepared by in-situ oxidative polymerization of aniline in the presence of CNT. To 100 mL of 0.1 M HCl solution, the CNTs (5 wt% of aniline) were dispersed by stirring and ultrasonic bath. After uniform distribution of CNTs, 1 g of aniline was added to this dispersion for proper adsorption of aniline monomer on CNTs and subsequently the solution of oxidant (1.45 g PPs in 100 mL 1 M HCl) was added to start the polymerization. The reaction mixture turned into greenish black which was filtered after 24 h, washed with water and ethanol and eventually dried at 100 °C for 6 h to get PANI@CNT. Pure PANI was also prepared similarly but without CNTs. For PANI@CNT/MnO_2_, similar methodology as of PANI@CNT was adopted but for the addition of oxidant step. Here a slight modification was done and to the dispersion of aniline and CNTs in 100 mL of 1 M HCl solution, a 100 mL dispersion of 0.2 g of MnO_2_ in 1 M HCl was added to affect the polymerization.

### 2.3. Characterization

The morphological and compositional analysis of MnO_2_, PANI, PANI@CNT and PANI@CNT/MnO_2_ was conducted by Field emission scanning electron microscopy (FESEM, JSM-7600F (JEOL, Tokyo, Japan)) and energy dispersive X-ray spectroscopy (EDS, Oxford Instruments, High Wycombe, UK). For the crystal structure and phase determination analysis, X-ray diffraction (XRD) was performed in 2θ range of 10°–90° (Rigaku, Ultima IV, Tokyo, Japan). Raman analysis was done by Thermo Fisher Scientific instrument (DXR Raman Microscope, Madison, WI, USA). For the elemental analysis and interactions between the constituents, X-ray photoelectron spectroscopy (XPS) (PHI 5000 Versa Probe II, Physical Electronics, Chanhassen, MN, USA) was performed at ~10^−10^ m bar.

### 2.4. Electrodes Development and Electrochemical Analysis

To develop supercapattery, the prepared composites were used as the positive and AC as the negative electrode. For the fabrication of electrodes, active materials (MnO_2_, PANI, PANI@CNT and PANI@CNT/MnO_2_) were coated by drop cast method on nickel foam of 1 × 1 cm^2^ area. Firstly, a slurry was prepared by mixing the active material (75%), AC (15 wt%), and PVdF (10 wt%) in NMP and was stirred at ambient conditions for 24 h in order to get a homogenous slurry. The slurry was coated on the nickel foam by drop casting and was subsequently heated at 90 °C for 12 h. The active material was loaded on all electrodes in the mass of ~5.00 mg.

An electrochemical workstation (Gamry Interface 1000 Instrument, Warminster, PA, USA) was utilized to examine the electrochemical behavior. A potential window of 0–0.5 V was used in cyclic voltammetry (CV) versus Ag/AgCl reference electrode. The current densities of 0.3 to 0.8 A g^−1^ at 0.5 V were used for galvanostatic-charge–discharge (GCD) studies and electrochemical impedance spectroscopy (EIS) was carried out in the range 0.01–100 kHz at a fixed AC voltage of 10 mV (RMS, root mean square potential amplitude). All electrochemical measurements were performed in 0.1 M KOH aqueous electrolyte.

## 3. Results and Discussion

### 3.1. Morphological Characterizations

**Figure 1:**
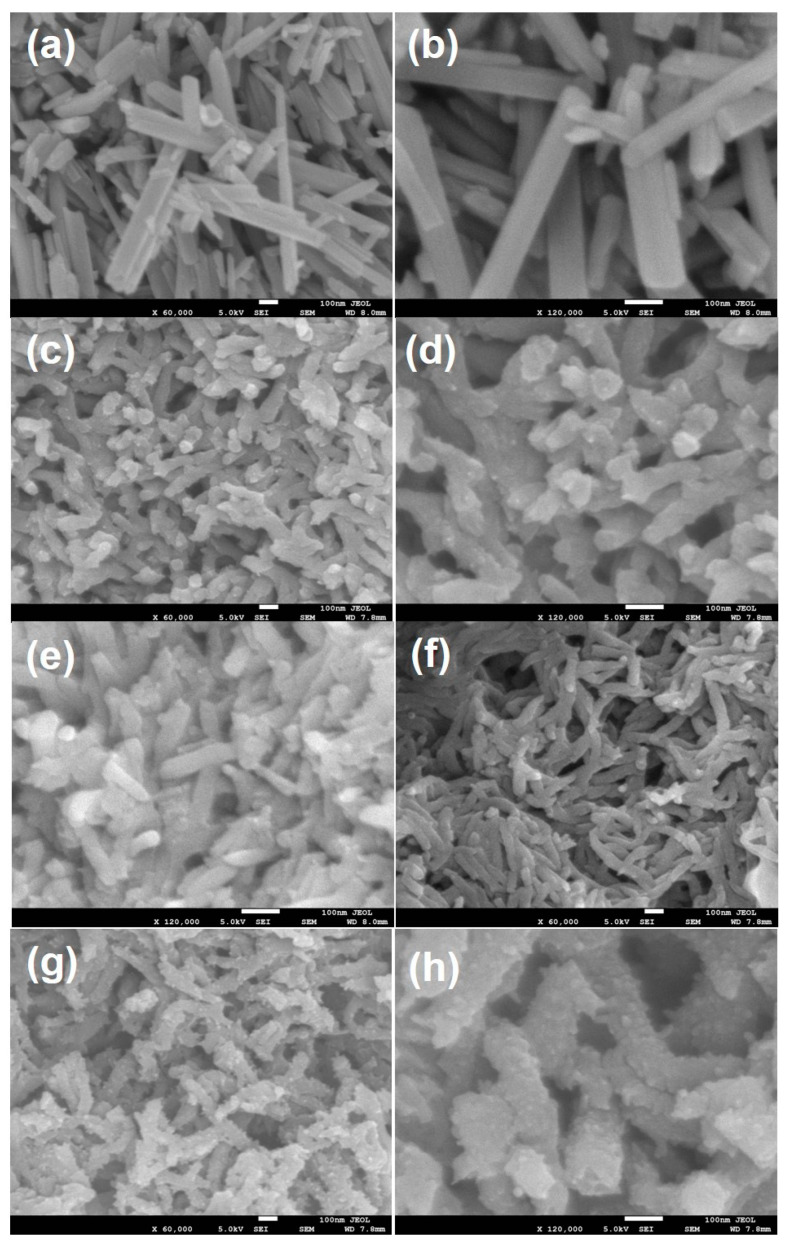
Field emission scanning electron microscopy (FESEM) images of (**a**,**b**) MnO_2_ nanorods, (**c**,**d**) polyaniline (PANI), (**e**,**f**) PANI@CNT and (**g**,**h**) PANI@CNT/MnO_2_ showing MnO_2_ and carbon nanotubes (CNTs) decorated/embedded in PANI fibrous network.

The SEM micrographs of MnO_2_, PANI, PANI@CNT and PANI@CNT/MnO_2_ are presented in Figure 1. In the case of MnO_2_, highly monodispersed MnO_2_ rods with shaped defined edges can be clearly seen (Figure 1a). The diameter of the rods varies from 60–120 nm while its length is in the range of 500–600 nm with many intermediate smaller sized rods also in the vicinity. It can be interpreted that the presence of many smaller particles on MnO_2_ or smaller sized rods may be due to the breakage of larger rods or due to the small amount of KMnO_4_ which failed to grow into rods [22]. Pure PANI showed fibrous morphology of diameter ~30–70 nm with some larger fibers of length exceeding 400 nm while some smaller sized rods are also seen (Figure 1b). The fibrous morphology of PANI is due to the fast polymerization of aniline during the rapid mixing technique as explained in our previous report [23]. In the case of PANI@CNT, similar morphology as in the case of PANI was observed with little or no observance of CNT owing to the surface coating of PANI over it (Figure 1c) [24]. The ternary composite PANI@CNT/MnO_2_ showed striking different morphology of PANI coated CNT/MnO_2_, with fibers showing interrupted or rough surface which is completely different to that of pure PANI and MnO_2_ (Figure 1d). It can be interpreted that the part of MnO_2_ which oxidizes aniline to PANI, dissolves or disintegrates during the genesis of PANI tubes leading to the breakage and rough outer surface of PANI. The rough interwoven fibrous and porous structure of PANI@CNT/MnO_2_ is expected to provide enhanced surface area and will provide channels for the transport of ions or charge carriers.

**Figure 2:**
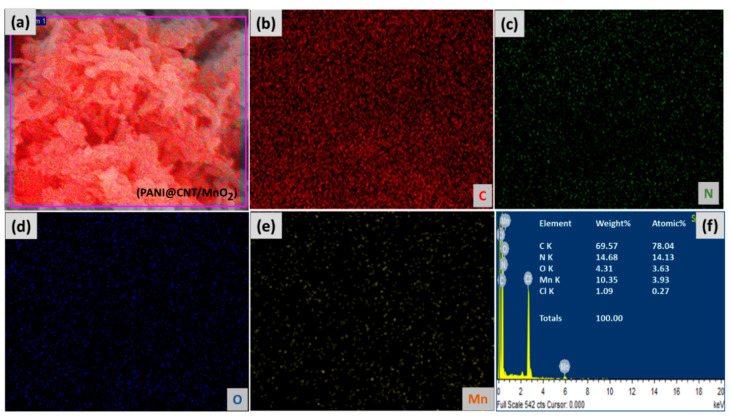
(**a**) Energy dispersive X-ray spectroscopy (EDS) elemental mapping of PANI@CNT/MnO_2_, (**b**) C, (**c**) N, (**d**) O, (**e**) Mn, and (**f**) compositional spectra of the PANI@CNT/MnO_2_ ternary composite.

The EDS analysis of PANI@CNT/MnO_2_ in Figure 2 shows the presence of C which corresponds to the carbon skeleton of PANI and CNT, the N corresponds to the imine group of PANI, while the presence of Mn and O confirms the presence of MnO_2_. The small amount of Cl confirms the acid doping of PANI@CNT/MnO_2_ composite.

The elemental mapping of PANI@CNT/MnO_2_ also showed the presence of C, N, O, Mn and Cl with homogenous dispersion which suggests the efficacy of the synthesis methodology (Figure 2).

### 3.2. X-ray Diffraction and Raman Studies

**Figure 3:**
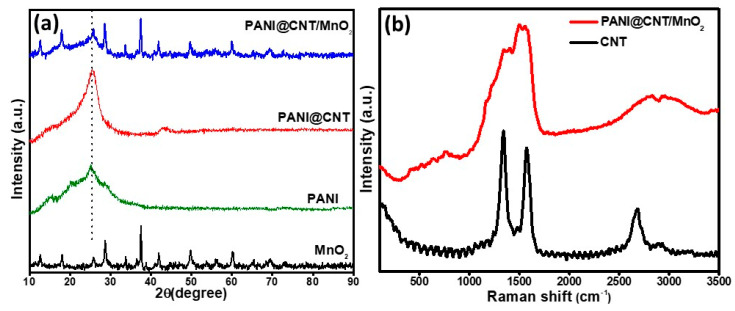
(**a**) X-ray diffraction (XRD) pattern of the MnO_2_, PANI, PANI@CNT binary and PANI@CNT/MnO_2_ ternary composites scanned in the 2θ range of 10–90° and (**b**) Raman spectra of the pure CNT and PANI@CNT/MnO_2_ ternary composite.

The XRD analysis of MnO_2_, PANI, PANI@CNT and PANI@CNT/MnO_2_ is presented in Figure 3a. In the case of MnO_2_, the prominent peaks at 2θ = 12.71°, 17.99°, 28.69°, 37.54°, 42.03°, 49.80°, 56.30° and 60.30° corresponds well to the crystal planes of α-MnO_2_ (JCPDS card PDF file no. 44–0141) [25]. PANI emeraldine salt showed a prominent peak at 2θ = 25.28° with a slight shoulder at *2θ* = ~15 and 20 corresponding to the 322, 121 and 113 planes respectively, suggesting that most of the PANI is ordered along these crystal planes [26]. The maximum sharpness of 322 plane peak suggests that most of the arrangement is periodicity perpendicular to the polymer chain [27]. PANI@CNT shows two peaks at 2θ = ~25.60° and 43° corresponding to the reflections due to the graphitic (0 0 2) and (1 0 0) planes [28]. The peak at 2θ = ~25° of CNT also coincided with the 322 peak of PANI, but in contrast to pure PANI, the XRD peak of PANI@CNT at 2θ = ~25° is much shaper and intense owing to the high crystallinity of CNTs. The ternary composite PANI@CNT/MnO_2_ shows all the reflections of PANI, CNT and MnO_2_ which suggest successful intercalation of the constituents. However, the peaks are of much reduced intensity which might be due to in-situ oxidation of aniline by MnO_2_ as it might result in the disruption of MnO_2_ crystals during its dissolution, coating of MnO_2_, PANI or by both over CNT.

The Raman spectra of CNT and PANI@CNT/MnO_2_ is presented in Figure 3b. Pure CNT shows a typical G band (derived from the graphite like mode) at 1580 cm^−1^. The defects or disorder induced D-band gap is situated at 1346 cm^−1^ [29]. After coating with PANI and MnO_2_ in PANI@CNT/MnO_2_, the spectra of PANI is dominant which confirms good coating of CNT with PANI [30].

### 3.3. X-ray Photoelectron Spectroscopy Studies

**Figure 4:**
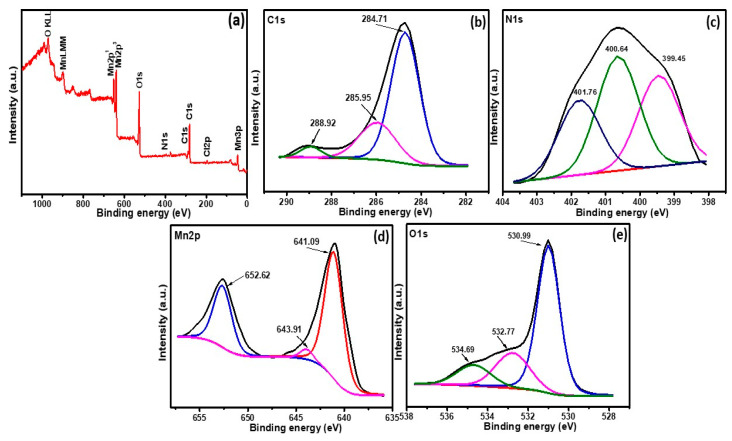
(**a**) A survey spectrum of PANI@CNT/MnO_2_ ternary composite, core-level XPS spectra of (**b**) C 1s, (**c**) N 1s, (**d**) Mn 2p and (**e**) O 1s.

The XPS analysis was done to study the composition and chemical states of PANI@CNT/MnO_2_ (Figure 4). The survey scan of PANI@CNT/MnO_2_ (Figure 4a) shows the presence of five major sets of peaks corresponding to C, N, O, Cl and Mn without the observance of any other impurities. The C1s peak corresponds to the residual carbon of the sample and carbon from the XPS instrument. The C1s peak (Figure 4b) can be deconvoluted into three peaks at 284.71, 285.85 and 288.92 eV corresponding to the aliphatic carbon C–C/C–H, C–O–C and O–C=O [31]. The C–O–C and O–C=O also represents some additional functionality of PANI and CNT which might acts as sites for interaction with each other or MnO_2_.

The N1s peak (Figure 4d) can be deconvoluted into three peaks at 399.45, 400.64 and 401.76 eV corresponding to the pyridinic nitrogen, pyrrolic nitrogen/pyridone nitrogen in association with oxygen functionality and quaternary nitrogen respectively [32]. In Mn*2p* spectra (Figure 4e) two peaks at ~638 and 649 eV with a separation gap of ~11 eV can be attributed to the Mn*2p3/2* and Mn*2p1/2* respectively. These values are in agreement with the previous reports and indicate +4 oxidation state of Mn [33]. This also suggests successful synthesis of MnO_2_ and also its state has not changed in the ternary composite. In the O*1s* spectra (Figure 4e) the 530.99 eV can be associated with the oxygen attached to Mn. The other bands at 532.77 and 534.69 eV is due to organic C=O or H–O–H and C=O, respectively [34]. The small amount of Cl in the survey scan is from HCl due to the acid doping of PANI.

### 3.4. Three Electrodes Electrochemical Studies of MnO_2_, PANI, PANI@CNT and PANI@CNT/MnO_2_

**Figure 5:**
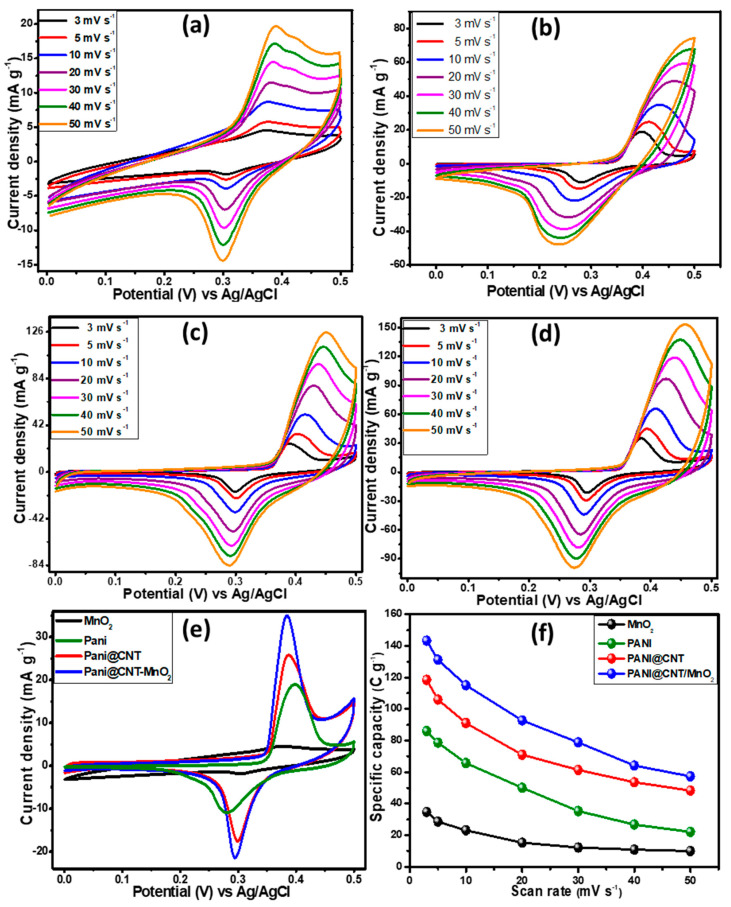
Cyclic voltammetry (CV) curves of (**a**) pristine MnO_2_ nanorods (**b**) fibrous PANI, (**c**) PANI@CNT binary composite, (**d**) PANI@CNT/MnO_2_ measured at varied scan rates, (**e**) CV curves to compare the electrochemical performance of all variants recorded at 3 mV s^−1^, and (**f**) scan rate vs. specific capacity plot for all variants.

Electrochemical studies of the as synthesized ternary composite and its variants were carried out in an alkaline medium (0.1 M KOH) in the potential range of 0 to 0.5 V using Ag/AgCl as reference and platinum wire as counter electrode through cyclic voltammetry (CV), galvanostatic charge–discharge (GCD) at various current densities (0.3–0.8 A g^−1^) and electrochemical impedance spectroscopy (EIS) in a standard three electrodes cell system. Figure 5 shows the electrochemical signature of MnO_2_, PANI, PANI@CNT and PANI@CNT/MnO_2_ at different scan rates from 3 to 50 mV s^−1^. The lowest and the highest scan rates demonstrate the stability and rate capability of the electrode materials [35]. In each case the redox peaks in the voltammograms can be observed, demonstrating the occurrence of faradic reaction at the electrode which is the property of a battery grade material [36]. Figure 5a shows the MnO_2_ CV curves at different scan rates. There is a linear increase in the current densities when the scan rates were increased showing the good electrochemical behavior and reached to the maximum current density of 19.88 mA g^−1^ at a scan rate of 50 mV s^−1^ as a standalone electrode material when the particles size is in the nano regime [37]. The metal oxides in their pristine forms tend to aggregate, which reduced the electrochemical active sites of the nanomaterials and ultimately affected the electrochemical activity of the electrode materials. In the case of PANI (Figure 5b), when scanned from 3 to 50 mV s^−1^, a maximum current density of 74.76 mA g^−1^ was observed because PANI is well known conducting polymer and its fibrous morphology is most conductive in nature compared to nanospheres and nanorods [38]. Figure 5c represents CV curves of PANI@CNT. Carbon nanotubes have excellent conductivity and thermal stability. Therefore, due to the additional effect the current density of PANI@CNT was increased to 126.37 mA g^−1^ at 50 mV s^−1^ compared to pure PANI. However, the maximum current density (154.84 mA g^−1^ @ 50 mV s^−1^) was achieved in the case of the ternary composite, PANI@CNT/MnO_2_ as demonstrated in Figure 5d, which could be due to the synergistic effect of CNT and MnO_2_ nanoparticles in the ternary composite [39]. Additionally, the porous nature of the ternary composite plays a pivotal role in the perforation of counter ions that help to enhance the conductivity of the active material. When the MnO_2_ nanorods are embedded in the polymer matrix the phenomena of de-aggregation occurred, which helped to expose the more electroactive site of the MnO_2_ nanoparticles. Due to the de-aggregation of MnO_2_ nanoparticles and addition of CNT in polymer matrix, the highest conductivity was achieved due to the synergistic effect of the contributing components in ternary composite as shown in comparison plot (Figure 5d). In all cases, there is a slight peak potential shift at high scan rates due to the asynchronous movement of the counter ions. These results are providing the evidence of useful strategy that a combination of three components (PANI, CNT, MnO_2_) in the form of composite by a facile synthesis protocol and porosity can produce enhanced electrochemical signature than the individuals. A comparison plot (Figure 5e) of all variants shows the systematic increase in the current densities. The specific capacity ($Q_{S}$) for all electrode materials from CV curves was calculated using the following Equation (1) [40]:

(1) $$Q_{S}=\frac{1}{m\upsilon }\int_{Vi}^{Vf}I\times VdV$$

where $Q_{S}$, *m*, *υ*, and integral part of the equation represent specific capacity (C g^−1^), active mass (g), scan rate (mV s^−1^) and area under the I-V curve respectively. The $Q_{S}$ values calculated at 3 mV s^−1^ for MnO_2_, PANI, PANI@CNT, and PANI@CNT/MnO_2_ are 34.71, 86.02, 118.47, and 143.26 C g^−1^, respectively. The specific capacity versus scan rates is plotted in Figure 5f. It could be concluded that specific capacity depleted at higher scan rate, but the ternary composite is the best sustaining electrode material even at high scan rate among other samples when tested under the same conditions.

**Figure 6:**
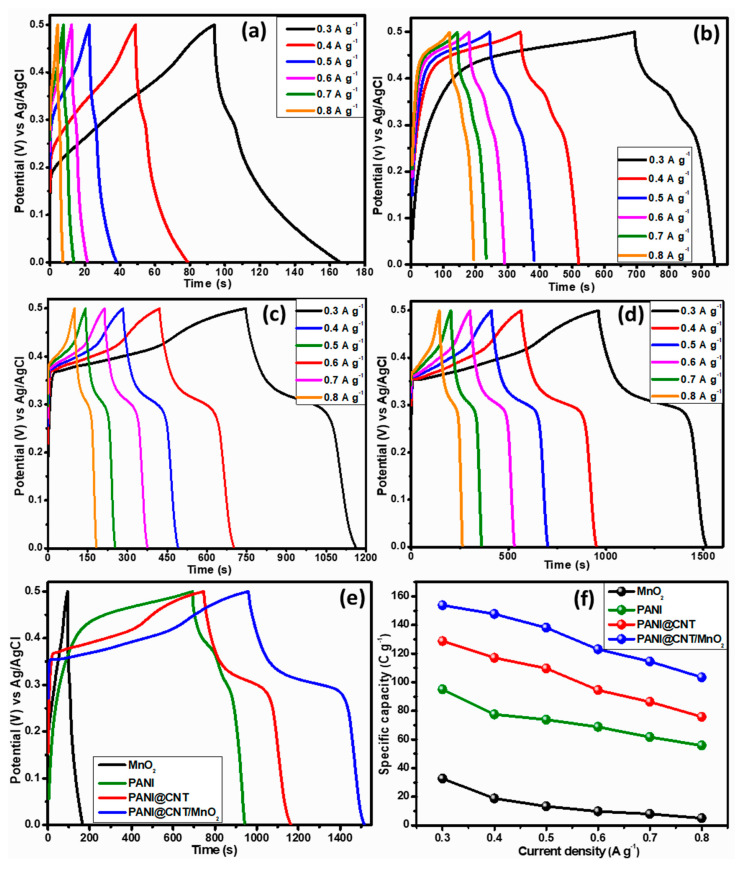
Galvanostatic-charge–discharge (GCD) curves at various current densities, (**a**) MnO_2_, (**b**) fibrous PANI, (**c**) PANI@CNT, (**d**) PANI@CNT/MnO_2_, (**e**) comparison curves for all four variants at a current density of 0.3 A g^−1^, and (**f**) current density vs. specific capacity plot of all four variants.

The GCD studies presented in Figure 6 were conducted at a fix potential of 0.5 V vs. Ag/AgCl at different current densities. It is obvious from the GCD curves (Figure 6a–d) that there is a systematic increase in the discharge time for all four variants, but the ternary composite takes a lead among all, as shown in the comparison plot (Figure 6e). The non-triangular GCD curves vouch the non-electrostatic behavior of the electrode materials. Similarly, $Q_{S}$ values were also calculated from GCD curves for all variants using the following Equation (2) [41]:

(2) $$Q_{S}=\frac{I\times \Delta t}{m}$$

where *I*, ∆*t* and *m* represent current density, discharge time and active mass in the equation, respectively. The obtained $Q_{S}$ values at current density 0.3 A g^−1^ are 32.98, 95.04, 128.73, and 153.82 C g^−1^ for MnO_2_, PANI, PANI@CNT, and PANI@CNT/MnO_2_, respectively. The specific capacity values at various current densities are plotted in Figure 6f. It is clear the ternary composite showed the highest specific capacity and better rate capability among all other variants.

**Figure 7:**
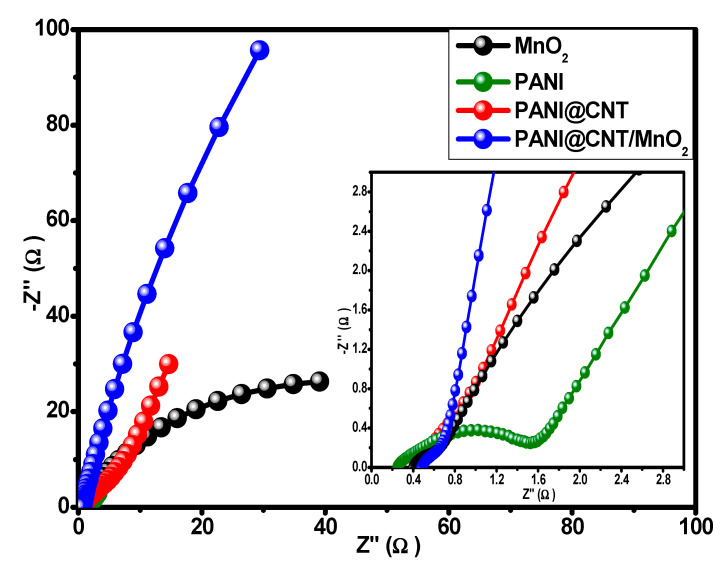
Nyquist plots of the MnO_2_ nanorods, fibrous PANI, PANI@CNT, and PANI@CNT/MnO_2_ ternary composite. Inset Figure 7 shows the electrochemical impedance spectroscopy (EIS) spectrum in high frequency region.

The EIS studies were conducted to analyze the intrinsic properties such as equivalent series resistance (*ESR*), charge transfer resistance (*R_ct_*) and Warburg impedance. EIS studies were conducted at an alternating voltage of 10 mV (RMS) in the frequency range of 0.01–100 kHz. The Nyquist plot for different variants are shown in Figure 7. The graph demonstrates that the ternary composite (0.49 Ω) has the least charge transfer resistance compared to PANI (1.40 Ω), MnO_2_ (0.54 Ω) and PAN@CNT (0.62 Ω) as well as most vertical line parallel to the imaginary axis (-*Z*″) showing the best charge storage capacity. Similarly, ESR values obtained for MnO_2_, PANI, PANI@CNT, and PANI@CNT/MnO_2_ are 0.45, 0.22, 0.38, and 0.48 Ω, respectively. The best performing electrode material was then selected for device fabrication. In this case, it is concluded from all above electrochemical studies that ternary composite, PANI@CNT/MnO_2_ is the best battery type electrode material which can be utilized for hybrid device assembly.

### 3.5. Fabrication and Characterization of Two Electrodes Assembly (PANI@CNT/MnO_2_//AC)

**Figure 8:**
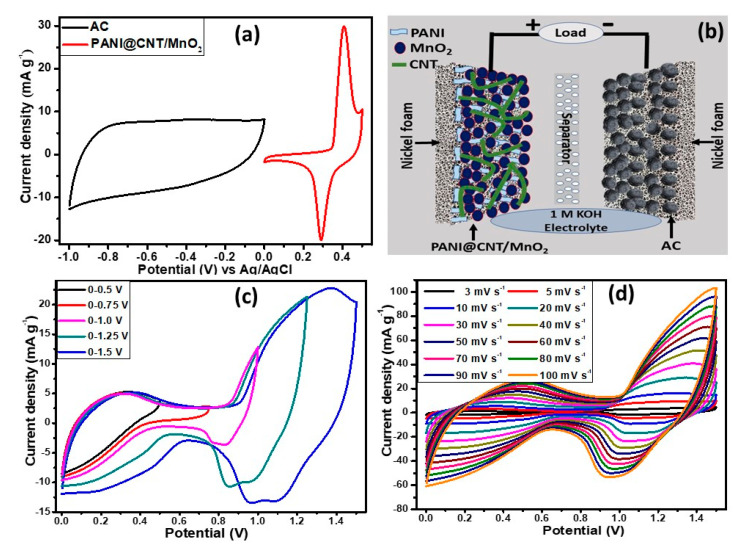
(**a**) Comparative cyclic voltammetry (CV) curves of PANI@CNT/MnO_2_ ternary composite and activated carbon (AC) electrodes performed in a three-electrode cell in KOH aqueous solution at a scan rate of 10 mV s^−1^; (**b**) graphical representation of assembled device; (**c**) CVs of PANI@CNT/MnO_2_//AC supercapattery measured at different potential windows; and (**d**) galvanostatic charge–discharge curves of PANI@CNT/MnO_2_//AC supercapattery at various current densities.

The two electrodes assembly was developed using a nickel foam coated ternary composite, PANI@CNT/MnO_2_ as positive electrode (battery-grade material) and activated carbon (AC) coated as negative electrode (capacitive material). Both electrodes were coupled together by placing porous membrane in between them. In this assembly two different electrodes were used, i.e., supercapacitor electrodes and battery type electrodes, known as supercapattery, which can store charge electrostatically and electrochemically. Before combining them together both were tested individually in three electrodes cell to know their working potential window and electrochemical signature. Figure 8a shows that the AC is capacitive electrode with a potential window of −1 to 0 V whereas ternary composite is pseudocapacitive with potential window, 0 to 0.5 V. Figure 8b shows the graphical demonstration of the assembled device in which positive potential is applied to battery type electrode whereas negative potential to capacitive electrode. The electrochemical studies (CV, GCD and stability) of two electrodes assembly were conducted in 0.1 M KOH in the potential range of 0 to 1.5 V.

**Figure 9:**
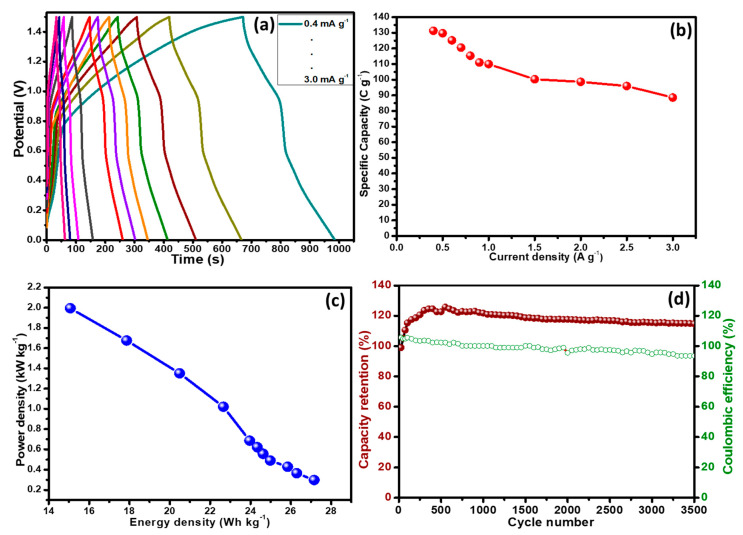
(**a**) GCD plot of PANI@CNT/MnO_2_ ternary composite for the assembled hybrid device, (**b**) current density versus specific capacity, (**c**) energy density versus power density change, and (**d**) cyclic performance and coulombic efficiency of the PANI@CNT/MnO_2_ ternary composite based hybrid device as a function of the number of cycles at a current density of 3.0 A g^−1^ in 0.1 M KOH.

The CV studies were conducted at different potential windows (0–0.5, 0–0.75, 0–1.0, 0–1.25 and 0–1.5 V) to verify the maximum working potential range as shown in Figure 8c. The assembled hybrid device then scanned at various rates from 3 to 100 mV s^−1^ at a fix potential window of 0 to 1.5 V as demonstrated in Figure 8d to analyze the rate capability. The shape of the CVs was well conserved even at higher scan rates thereby representing the marvelous rate capability. The shape of the CVs is neither rectangular nor humpy, showing the contribution of both energy storage mechanism, i.e., non-faradaic (capacitive) and faradaic (diffusive). In GCD studies (Figure 9a), similar trend was observed because the curves were not completely rectangular but each one has little shoulder. Here again, the assembled hybrid device demonstrating the charge storage mechanism is contributed by both capacitive and diffusive processes. The $Q_{S}$ values were calculated form the GCD curves for the assembled hybrid device at various current densities using Equation (2).

The highest specific capacity of 131.27 C g^−1^ was found at current density of 0.4 A g^−1^ for the assembled hybrid device as shown in Figure 9a.

The energy density (*E*) and power density (*P*) are the major parameters to assess the working of an energy storage device which were calculated using the following Equations (3) and (4):

(3) $$E\left(\mathrm{Wh/kg}\right)=\frac{\Delta V\times Q_{S}}{2\times 3.6}$$

(4) $$P\left(\mathrm{W/kg}\right)=\frac{E\times 3600}{\Delta t}$$

where $Q_{S}$, ∆*V*, and ∆*t* represent the specific capacity (C g^−1^), potential window (*V*) and discharge time (*s*) of the hybrid device, respectively. The obtained values of energy density and power density are plotted in Figure 9c. The maximum energy density (27.17 Wh kg^−1^) was obtained at a current density of 0.3 A g^−1^ with the corresponding power density of 298.00 W kg^−1^. An inverse relation was noticed between energy density and power density. When the power density was stepped up from 298.00 to 1995.00 W kg^−1^, energy density depleted down from 20.47 to 15.07 W kg^−1^. These results proved the excellent working of the hybrid assembled device using the novel ternary composite when assessed based on energy density, power density and specific capacity. Another important parameter for energy storage devices is the stability test. The assembled hybrid device was subjected to charge–discharge cycles for over 3500 cycles. Figure 9d shows the initial increase in the specific capacity which is due to the activation of polymer composite. A similar trend has been reported in many other articles, especially, where conducting polymers were used in the composites [3,42]. However, after about ~1000 cycles the stability in the specific capacity was observed, and finally at the end of 3500 cycles, excellent (119.00%) specific capacity with coulombic efficiency of 93.50% was demonstrated by the assembled hybrid device. These results advocate the successful synthesis of porous ternary composite in which three components were combined by a simple hydrothermal method coupled with in-situ oxidative polymerization to achieve synergistic/additional electrochemical effect and subsequently utilized as a battery type electrode material in supercapattery. We compared our results with the reported literature on polyaniline based composite electrode materials, which is presented in Table 1.

## 4. Conclusions

In summary, PANI@CNT/MnO_2_ nanocomposites was prepared by the combination of in-situ oxidative polymerization and hydrothermal reaction. The FESEM images showed fibers with interrupted or rough surfaces, which is expected to provide an enhanced surface area and facilitate in the transport of ions or charge carriers. The XRD and XPS analyses of PANI@CNT/MnO_2_ showed peaks corresponding to the MnO_2_, CNT, and PANI, thereby suggesting the efficacy of the synthesis methodology. The electrochemical studies were carried out to analyze the voltammetric character through a typical three electrode system and applied voltages ranging from 0 to 0.5 V. PANI@CNT/MnO_2_ battery type ternary nanocomposite showed highest current density of 154.84 mA g^−1^ @ 50 mV s^−1^ and specific capacity of 143.26 F g^−1^ at 3 mV s^−1^ in comparison to 34.71 for MnO_2_, 86.02 for PANI and 118.47 C g^−1^ for PANI@CNT. The galvanostatic charge–discharge studies showed that there is systematic increase in the discharge time for all four variants but PANI@CNT/MnO_2_ ternary composite demonstrated the best electrochemical performance in terms of longer discharge duration. The specific capacity from GCD curves at current density 0.3 A g^−1^ were 32.98, 95.04, 128.73, and 153.82 C g^−1^ for MnO_2_, PANI, PANI@CNT, and PANI@CNT/MnO_2_, respectively. The Nyquist plots for the performed EIS study showed that PANI@CNT/MnO_2_ has the smallest radius among all other electrode materials at high frequencies with a more vertical line at low frequencies parallel to the imaginary axis. This confirms that PANI@CNT/MnO_2_ battery type material possesses highest capacity of charge storage and least resistance against the charge transfer. The enhanced performance of PANI@CNT/MnO_2_ can be attributed to the high thermal and electrical conductivity of the constituents which assist in smooth conductive path for the transportation of charge carriers along MnO_2_, CNT and PANI. Apart from this, the assembled supercapattery—consisting of the battery type ternary composite as a positive and an activated carbon as the negative electrode—delivered the highest specific capacity of 131.27 C g^−1^ which was found at a current density of 0.4 A g^−1^. The maximum energy density of 27.17 Wh kg^−1^ was obtained at a current density of 0.3 A g^−1^ with the corresponding power density of 298.00 W kg^−1^. The cyclic stability of the fabricated device showed that the PANI@CNT/MnO_2_ battery type ternary composite retained 119% capacitance even after 3500 cycles. The high specific capacitance combined with excellent cyclic stability projects PANI@CNT/MnO_2_ to be a promising battery type material for high performance supercapattery.

## Figures and Tables

**Table 1:** Polyaniline based composite electrode materials for supercapacitors.

Electrode Material	Electrolyte	Specific Capacity	Energy Density	Cycle Life % Retention/Cycles	Ref.
Graphene/polyaniline nanosheets	6 M KOH	261.4 F g^−1^ at 100 mA g^−1^		61.67%/500 cycles	[43]
CoFe_2_O_4_/reduced graphene oxide/polyaniline composite	1 M KOH	9 mF m^−1^ at 1 mA	270 × 10^−8^ Wh cm^−1^	87%/1000 cycles	[44]
Cobalt hydroxide/polyaniline hybrid nanostructure	1 M NaOH	215 F g^−1^ at 10 mV s^−1^	×	60%/1000 cycles	[45]
CoFe_2_O_4_/reduced graphene oxide/polyaniline composite	1 M KOH	9 mF m^−1^ at 1 mA	270 × 10^−8^ Wh cm^−1^	87%/1000 cycles	[44]
Manganese dioxide-polyaniline composite	Polyvinyl alcohol/KOH gel	129.2 F g^−1^ at 0.5 A g^−1^	22.3 Wh kg^−1^	89%/5000 cycles	[46]
NiCo_2_S_4_/polyaniline nanosheets	Polyvinyl alcohol/KOH gel	152.06 F g^−1^ at 1 A g^−1^	54.06 Wh kg^−1^	91.1%/2000 cycles	[47]
Acetylene black-manganese cobaltite- polyaniline composite	1 M KOH	0.35 F cm^−2^ at 1 mA cm^−2^	18.203 Wh kg^−1^	90%/3000 cycles	[48]
Strontium oxide/graphene/polyaniline ternary composite	1 M KOH	151.66 C g^−1^	33.8 Wh kg^−1^	80%/3000 cycles	[49]
Metal organic framework (MOF)/polyaniline composites	1 M KOH	162.5 C g^−1^ at 0.4 A g^−1^	23.2 Wh kg^−1^ at 1 A g^−1^	146%/3000 cycles	[35]
Co_3_O_4_/Ag/polyaniline ternary composites	0.1 M KOH	262.62 C g^−1^ at 3 mV s^−1^	14.01 Wh kg^−1^ at 0.2 A g^−1^	121.03%/3500 cycles	[50]
polyaniline@CNT/MnO_2_ ternary composites	0.1 M KOH	131.27 C g^−1^ at 0.4 A g^−1^	27.17 Wh kg^−1^ at 0.3 A g^−1^	119%/3500 cycles	Our work
